# Characterisation of serum progesterone and progesterone-induced blocking factor (PIBF) levels across trimesters in healthy pregnant women

**DOI:** 10.1038/s41598-020-59452-y

**Published:** 2020-03-02

**Authors:** Mark Kit Lim, Chee Wai Ku, Thiam Chye Tan, Yin Hao Justin Lee, John Carson Allen, Nguan Soon Tan

**Affiliations:** 1 Lee Kong Chian School of Medicine, Nanyang Technological University, 11 Mandalay Road, Singapore, 308232 Singapore; 2 KK Women’s and Children’s Hospital, 100 Bukit Timah Road, Singapore, 229899 Singapore; 3 Centre for Quantitative Medicine, Duke-NUS Medical School, 8 College Road, Singapore, 169857 Singapore; 4 School of Biological Sciences, Nanyang Technological University, 60 Nanyang Drive, Singapore, 637551 Singapore

**Keywords:** Predictive markers, Prognostic markers, Pregnancy outcome, Endocrine reproductive disorders, Endocrine reproductive disorders

## Abstract

Progesterone-induced blocking factor (PIBF), which plays an important role in maintaining healthy pregnancies, has shown great promise as a prognostic biomarker for threatened miscarriage. To better characterise the physiological trends of progesterone and PIBF, we analysed serum progesterone and PIBF concentrations in healthy non-pregnant and pregnant women across trimesters. We saw increasing concentrations of progesterone and PIBF in pregnant women with advancing trimesters. The serum progesterone and PIBF percentiles across gestational age in healthy pregnancies can be used as a guide for the formulation of reference ranges. We also demonstrated a significant positive correlation between progesterone and PIBF levels. This study demonstrates increasing progesterone and PIBF concentrations in later trimesters and underscores the importance of progesterone and PIBF in healthy pregnancies. Characterisation of progesterone and PIBF across gestational age in healthy pregnant women may help to prognosticate pregnancy viability and support further research into the importance of progesterone and PIBF in the maintenance of healthy pregnancies.

## Introduction

### Progesterone and progesterone-induced blocking factor (PIBF) are important in the maintenance of healthy pregnancies

Progesterone-induced blocking factor (PIBF), a protein comprising of 757 amino acid residues with a predicted molecular mass of 89 kDa, is important in the maintenance of human pregnancy and the progesterone dependent immunomodulation by the mother^1^.

Progesterone has been established to inhibit myometrial contractility, and progesterone withdrawal has been linked to the start of labour^2,3^. In addition, progesterone has been posited to play a crucial role in regulating the maternal immune response and preventing rejection of the fetal semi-allograft, which is mediated through PIBF^4,5^. During pregnancy, lymphocytes expressing progesterone receptors release PIBF in the presence of progesterone^4^.

PIBF is critical in supporting healthy pregnancies. This is supported by evidence that PIBF has been found to be depressed in women at risk of premature pregnancy termination and preterm births^6–9^. A Th2 cytokine profile is important in maintaining pregnancy, as evidenced by a reduced Th1/Th2 ratio in women with healthy pregnancies compared to women with complications during pregnancy or women who are not pregnant^10–12^. PIBF helps to inhibit the activity of natural killer (NK) cells and tilts the cytokine secretion profile in favour of Th2 type cytokine production^7,13–15^. Injections of potent interferon inducers and NK cell activators in murine models enhance fetal loss^16^, while treating spleen cells with high NK activity with PIBF nullifies the destructive effect of NK cells on the fetus^17^. A study reported that the use of anti-NK or anti-natural cytotoxic antibodies led to the reversal of the phenomenon of fetal resorption in mice with depleted PIBF, supporting the importance of PIBF in modulating NK cell activity and thereby ensuring healthy pregnancies^18^. PIBF also helps to inhibit phospholipase A2 which decreases the synthesis of prostaglandins from arachidonic acid, contributing to decreased IL-12 production and cytotoxic NK activity^1,19^. PIBF may also play critical roles in controlling trophoblast invasion through the suppression of pro-invasive genes^20,21^.

### Progesterone and progesterone-induced blocking factor (PIBF) are potential promising biomarkers for predicting pregnancy viability

Spontaneous miscarriage, occurring in 15 to 20% of all pregnancies, is the most common cause of vaginal bleeding in early pregnancy^22,23^. Vaginal bleeding affects a significant proportion of women (16% to 25%) in the first trimester of pregnancy^24^. The presence of vaginal bleeding is associated with a 2.6 times higher incidence of fetal loss^25^, and causes substantial anxiety and stress for the expectant mother^24,26^.

Threatened miscarriage is defined as vaginal bleeding with a closed cervix, in the first 20 weeks of a viable intrauterine pregnancy^22^. Accurate prediction of the outcome of threatened miscarriage in women is important in identifying women at high risk of eventual miscarriage and for providing timely advice and care to this high risk group. A plethora of miscarriage biomarkers, including human chorionic gonadotropin (hCG), progesterone, cancer antigen 125 (CA-125), inhibin A, activin A, pregnancy-associated plasma protein-A (PAPP-A) and kisspeptin have been proposed as tools to assess pregnancy viability^27–29^.

Many studies have shown that low serum progesterone is associated with threatened miscarriage. Our research group has validated the use of a serum progesterone cut-off value of <35 nmol/L in predicting subsequent miscarriage in women presenting with threatened miscarriage^6,30^. Given progesterone’s importance in maintaining healthy pregnancies, and PIBF’s pivotal role in maintaining a favourable immune environment for the semi-allogeneic fetus as a downstream effector protein of progesterone, both progesterone and PIBF are promising biomarkers for predicting pregnancy viability.

### Reference ranges for progesterone and progesterone-induced blocking factor (PIBF) are essential to supporting the use of progesterone and PIBF as biomarkers for predicting pregnancy viability

There is currently no generally accepted reference range, threshold or benchmark for normal serum PIBF levels across trimesters relative to serum progesterone in healthy pregnant women. The primary aim of this study is to characterise normal serum progesterone and PIBF values with advancing trimesters. This would enhance the potential of progesterone and PIBF in predicting pregnancy viability and aid in the development of the use of progesterone and PIBF levels as part of a resource saving and easy-to-use risk assessment tool for the identification and management of pregnant women at risk of miscarriage.

## Results

### Participants

183 women met the inclusion criteria and participated in the study. Of this group, 46 healthy non-pregnant women, and 47, 48 and 42 healthy pregnant women in the first, second and third trimesters respectively were recruited. All pregnant participants had successful term deliveries without complications.

### Maternal characteristics

Demographic variables collected from the participants (gestational age by ultrasound scan at recruitment, maternal age, body mass index (BMI), history of previous miscarriage, current smoker, and current alcohol drinker) were compared across the 4 subgroups (Table 1). Continuous variables were analysed with one-way ANOVA, while categorical variables were analysed using the chi-square test. Gestational age by ultrasound scan was significantly different among the 4 subgroups of participants, demonstrating the effective trimester segmentation of subgroups. Maternal age, BMI and history of previous miscarriages were comparable across the subgroups supporting the effectiveness of randomisation, while the proportion of current smokers and the proportion of current alcohol drinkers were predictably negligible in the healthy pregnant group relative to the non-pregnant group.

**Table 1:** Demographic data and serum biological markers of participants.

	Non-pregnant (n = 46)	First trimester (n = 47)	Second trimester (n = 48)	Third trimester (n = 42)	p value
**Demographic data**					
Gestational age by ultrasound scan at recruitment, mean ± SD (weeks)	NA	9.89 ± 1.17	22.5 ± 4.48	30.2 ± 2.77	**<0.0001**^**a**^
Maternal age, mean ± SD (years)	31.4 ± 5.99	29.6 ± 4.20	30.6 ± 3.52	31.8 ± 5.19	0.149^a^
Body mass index (BMI), mean ± SD (kg/m^2^)	26.7 ± 7.17	24.5 ± 5.33	24.7 ± 4.64	26.3 ± 4.10	0.133^a^
Previous miscarriage (%)	10.9	17.0	16.7	19.0	0.639^b^
Current smoker (%)	15.2	0	0	0	**<0.0001**^**b**^
Current alcohol drinker (%)	32.6	0	2.08	0	**<0.0001**^**b**^
**Serum biological markers**					
Progesterone, mean ± SD (nmol/L)	11.7 ± 17.0	67.2 ± 23.5	184 ± 81.0	296 ± 104	**<0.0001**^**a**^
[Progesterone, 95% CI of mean]	[6.65 to 16.8]	[60.3 to 74.1]	[161 to 208]	[263 to 328]	
Progesterone-induced blocking factor (PIBF), mean ± SD (ng/ml)	213 ± 185	612 ± 315	1100 ± 243	2510 ± 648	**<0.0001**^**a**^
[PIBF, 95% CI of mean]	[158 to 268]	[520 to 705]	[1030 to 1170]	[2300 to 2710]	

^a^denotes variables analysed with one-way ANOVA, while ^b^denotes variables analysed with the chi-square test.

### Serum progesterone and PIBF

**Figure 1:**
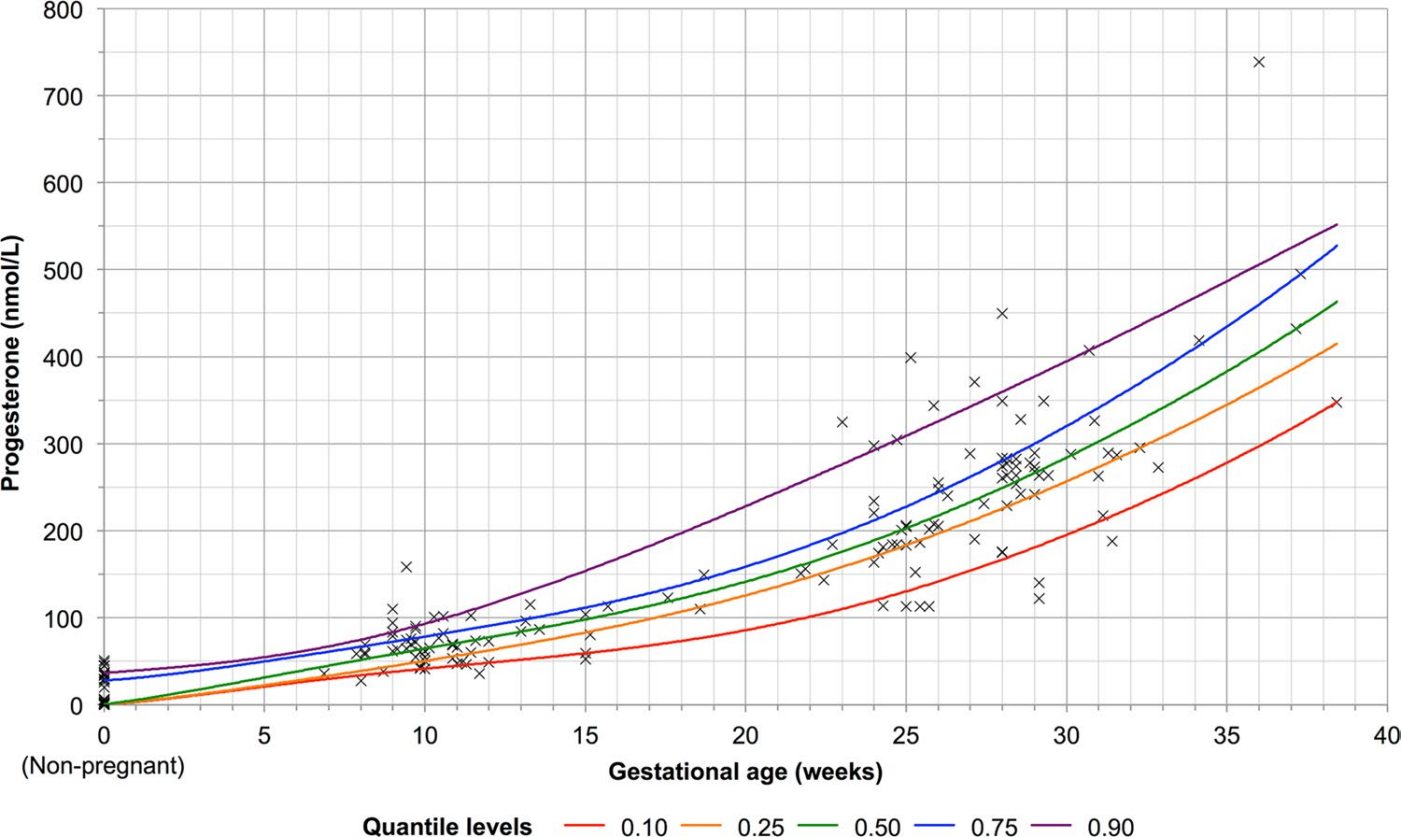
Quantile regression of progesterone (nmol/L) against gestational age (weeks). Individual plots represent individual data points. Plots at gestational age of 0 weeks represent data from the non-pregnant group.

Serum progesterone increased with advancing trimesters and increasing gestational age (Table 1 and Fig. 1). Serum progesterone was the lowest in healthy non-pregnant women (11.7 nmol/L, 95% CI 6.65–16.8 nmol/L), increasing from the first trimester (67.2 nmol/L, 95% CI 60.3–74.1 nmol/L), to the second trimester (184 nmol/L, 95% CI 161–208 nmol/L) and the third trimester (296 nmol/L, 95% CI 263–328 nmol/L).

**Figure 2:**
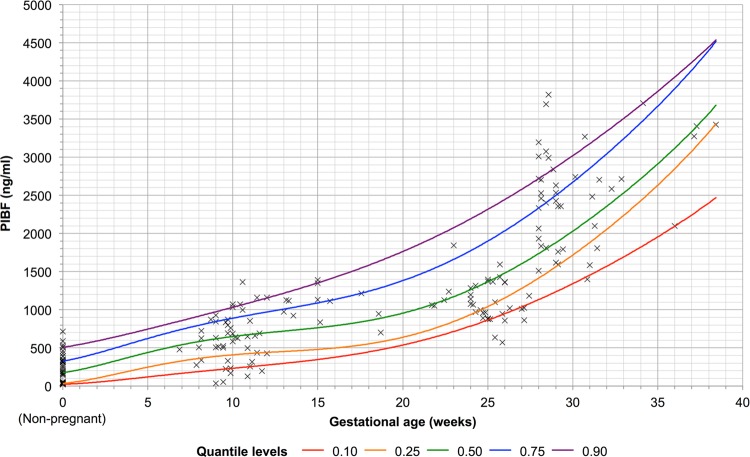
Quantile regression of progesterone-induced blocking factor (PIBF) (ng/ml) against gestational age (weeks). Individual plots represent individual data points. Plots at gestational age of 0 weeks represent data from the non-pregnant group.

Similarly, serum PIBF increased with advancing trimesters and increasing gestational age (Table 1 and Fig. 2). Serum PIBF was the lowest in healthy non-pregnant women (213 ng/ml, 95% CI 158–268 ng/ml), increasing from the first trimester (612 ng/ml, 95% CI 520–705 ng/ml), to the second trimester (1100 ng/ml, 95% CI 1030–1170 ng/ml) and the third trimester (2510 ng/ml, 95% CI 2300–2710 ng/ml).

**Figure 3:**
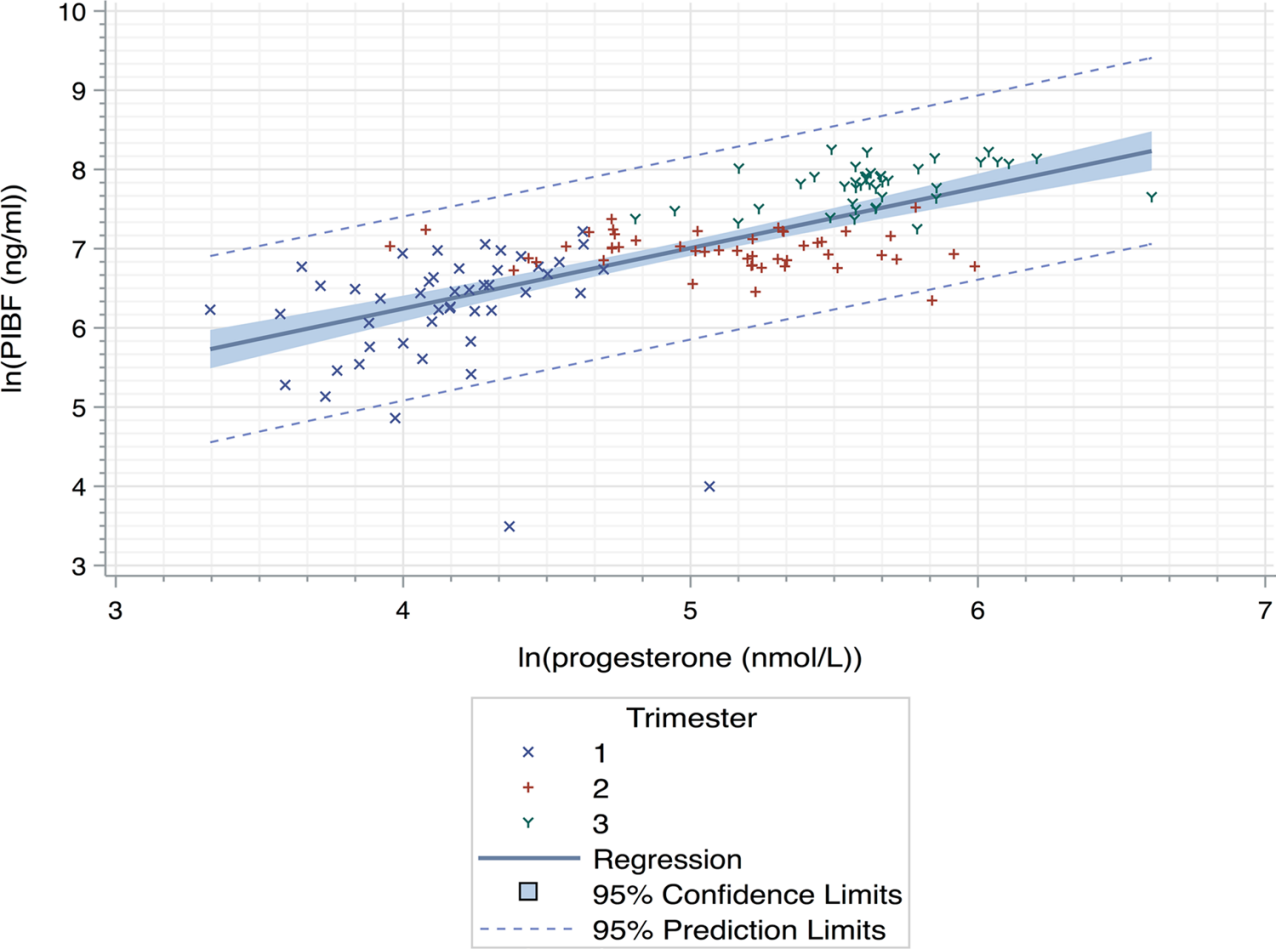
Linear regression of ln(progesterone-induced blocking factor (PIBF) (ng/ml)) on ln(progesterone (nmol/L)) for pregnant women. The equation of the line is y = 0.764 x + 3.19 (r^2^ = 0.474, p < 0.0001). Pearson correlation coefficient is r = 0.688 (p < 0.0001). Individual plots represent individual data points.

Linear regression of ln(PIBF) against ln(progesterone) revealed a significant positive linear relationship between the two variables with equation y = 0.764 x + 3.19 (r^2^ = 0.474, p < 0.0001) and Pearson correlation coefficient r = 0.688 (p < 0.0001) (Fig. 3). Data from the non-pregnant group were excluded from the linear regression, as our study sought to investigate the effect of increasing progesterone on PIBF in healthy pregnancies.

### Correlation of serum progesterone with BMI

**Figure 4:**
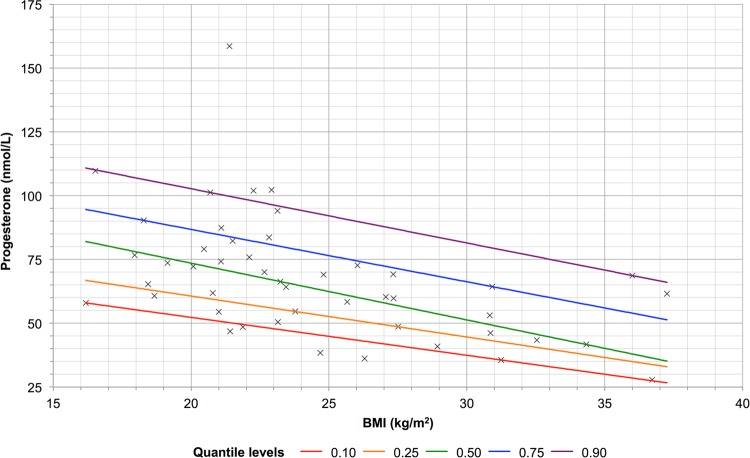
Quantile regression of progesterone (nmol/L) against body mass index (BMI) (kg/m^2^) for pregnant women in the first trimester. Individual plots represent individual data points.

A decrease in progesterone was seen with increasing BMI in pregnant women in the first trimester (Fig. 4). This relationship did not extend beyond the first trimester, and was not discerned in pregnant women in the second and third trimester (data not shown). There was no discernable relationship between PIBF and BMI (data not shown).

## Discussion

Both serum progesterone and PIBF increased with advancing trimesters and increasing gestational age. The increase in PIBF concentration showed a significant positive correlation with the increase in progesterone concentration. Our research group has previously described a linear increase in progesterone levels from 5 to 13 weeks of gestation in healthy pregnancies^31^, and this study further supports the crucial role of progesterone in healthy pregnancies.

This is one of the first prospective studies characterising the distribution of serum PIBF across trimesters in relation to progesterone in healthy pregnant women. There has been considerable interest in the development of reference ranges for serum PIBF across gestational age in relation to progesterone, which is PIBF’s physiological precursor. The serum progesterone and PIBF percentiles across gestational age can guide the development of reference ranges for serum progesterone and PIBF across gestational age, and contribute to a prediction of pregnancy viability.

A decrease in progesterone with increasing BMI above the normal range was seen in healthy pregnant women in the first trimester. This is consistent with another study showing a significant correlation between maternal obesity and low serum progesterone (defined as <35 nmol/l) in the first trimester^32^. Probable mechanisms include the association of obesity with a secondary central decrease in luteinising hormone leading to reduced progesterone production and premature luteolysis^33^, the deleterious effects of adipocytokines on corpus luteum function^34,35^, and an effectively lower serum progesterone concentration secondary to the pharmacokinetic effect of an increased progesterone distribution in adipose tissues due to the high lipid solubility of sex steroids^32^. These may support weight management and optimisation during the pre-conception period as possible means of increasing the chances of a successful pregnancy.

This study affirms previous studies outlining the importance of PIBF in pregnancy. PIBF modulates crucial effector functions, including shifting cytokine synthesis and cytotoxic cell activity towards a more immunotolerant state^1,9^. CD4+ and CD8+ T cells have been found to respond to progesterone in a dose dependent manner, causing marked changes in T-cell cytokine secretions at sites nearer to the fetus^36^. This dose dependent relationship between progesterone and PIBF is evidenced by the significant positive correlation between the two biomarkers seen in this study. The correlation is consistent with other studies that demonstrate increases in PIBF caused by progesterone^37^.

Characterisation of PIBF in healthy pregnancies across gestational age is anticipated to aid further investigations and assessments into its utility as a predictive tool for pregnancy viability. A potential advantage of PIBF over progesterone as a biomarker is that unlike progesterone, which undergoes reduction and glucuronidation before being excreted in different isomers, PIBF is excreted intact into the urine and can therefore be measured non-invasively^38^. In addition, unlike progesterone, which has been shown to decrease with increasing BMI, PIBF, a non-lipophilic protein, may not exhibit significant pharmacokinetic distribution in adipose tissues and hence may not be affected by BMI. Moreover, PIBF1 cDNA shares little amino acid homology with other known proteins^39^. These characteristics of PIBF enhance PIBF’s appeal as a specific target to predict pregnancy outcomes.

PIBF concentrations, which are detectable and quantifiable in the serum and urine of pregnant women, may have utility for predicting pregnancy outcomes^5^. PIBF concentrations in urine samples have been shown to continually increase until the 37^th^ gestational week, after which PIBF concentrations fall^38^. One study suggested a trend indicating higher miscarriage rates when PIBF is absent at 3 to 5 weeks of seemingly normal pregnancies^40^, while another study demonstrated a positive correlation between PIBF and successful conception^41^. This study, along with previous studies, reaffirms the critical role of PIBF in maintaining healthy pregnancies, and postulates the potential of PIBF as an effective screening tool for threatened miscarriage.

PIBF levels can be compared to progesterone levels in pregnant women to assess the degree of progesterone-dependent immunomodulation, which may provide valuable prognostic and therapeutic options for the mother. If PIBF is abnormally low compared to progesterone, it may indicate relative lymphocytic insensitivity to progesterone through impaired receptor stimulation or defective PIBF production in progesterone receptor positive lymphocytes, even in the presence of progesterone. The downregulation of the progesterone receptors and their downstream effector PIBF has been described in women with preterm deliveries, shorter gestational periods and negative pregnancy outcomes, and has been correlated with a dominant Th1 and pro-inflammatory state^42^. Progesterone concentrations at the extremities of the body are lower than concentrations at the maternal-fetal interface, which may enhance the importance of lymphocytic sensitivity to progesterone at the body’s peripheries in determining the eventual immunomodulatory effects of progesterone^1,43^. Correlating the progesterone and PIBF levels in the body’s peripheries may identify women at risk of miscarriage, and women who may benefit most from immunotherapy.

PIBF is recognised as a potential therapeutic agent or marker in lymphocyte immunotherapy for the reduction of miscarriage risk. Given that current research has pointed towards an active recognition and response to fetal antigens by the maternal immune system during pregnancy^44^, and the concomitant deviation of the maternal immune system towards that of tolerance as evidenced by the key role of regulatory T cells in healthy pregnancies^45^, lymphocyte immunotherapy may aid in supporting the development of immunological tolerance to the semi-allogeneic fetus. This therapy may be instrumental in treating recurrent spontaneous abortions, given that Th1 cytokines and NK cell over-activity are said to be major alloimmune drivers of this condition^46^. A meta-analysis has found that lymphocyte immunotherapy shows promise in improving in the live birth rate of women with unexplained recurrent spontaneous abortions^47^. Another study has demonstrated the increased lymphocytic expression of PIBF after lymphocytic immunisation using the male partner’s lymphocytes, pointing towards the utility of PIBF as a biomarker or active agent in alleviating miscarriage risk^48^. The findings of this study may provide guidance in PIBF dosing and therapeutic drug monitoring, if needed in the formulation of a safety profile and the elucidation of mechanisms behind such treatments.

Beyond obstetrics, PIBF may be relevant in oncology. PIBF has been described to suppress the immune response towards tumour cells, has been reported to be a positive regulator of tumour spread in primary lung and ovarian cells, and has been found to be expressed in the cytoplasm and nucleus of choriocarcinoma and primary tumour cells^49^. Furthermore, PIBF has been found to be expressed in various cancers, including glioblastoma multiforme, astrocytomas, and leukaemias, contributing to uncontrolled tumour proliferation and potential evasion of immune surveillance^50–52^. PIBF has also been reported to downregulate E-cadherin expression, possibly interfering with cell-cell adhesion mechanisms and increasing extracellular matrix degradation by matrix metalloproteinase (MMP), thus contributing to cancer invasion^49,53^. Therefore, beyond PIBF’s potential utility as a biomarker for predicting pregnancy viability, characterisation of normal PIBF values in healthy women may contribute to future research and development of PIBF as a potential cancer biomarker and therapeutic target used to monitor the degree of cancer control and augment targeted cancer therapy.

Despite the potential utility of progesterone and PIBF as biomarkers for predicting pregnancy viability, this study is limited by a relatively small sample size and its design as a cross-sectional study. Further studies, including longitudinal studies, must be conducted with larger sample sizes and in different populations to validate the reference ranges for progesterone and PIBF across gestational age. A previous pilot study had explored a “best subset” 3-factor model comprising of progesterone, fetal heart and BMI for the prediction of spontaneous miscarriage^6^. Future studies can formulate, evaluate and validate further predictive models for pregnancy viability that include progesterone and PIBF, along with other relevant demographic factors, imaging findings and laboratory markers. Additionally, current kits used for PIBF quantification exhibit high variability. Development of new kits with better sensitivity and affinity for PIBF will enable more accurate quantification of PIBF, and will pave the way for the wider use of PIBF.

## Conclusion

This study underscores the importance of progesterone and PIBF in healthy pregnancies, with increasing progesterone and PIBF concentrations seen in advancing trimesters and increasing gestational age. The characterisation of progesterone and PIBF levels across gestational age in healthy pregnancies and in healthy non-pregnant women, along with a significant positive correlation of progesterone and PIBF, can aid in the formulation of normal reference ranges for progesterone and PIBF, may help to prognosticate pregnancy viability, and can support further research into the importance of progesterone and PIBF in the maintenance of healthy pregnancies.

## Materials and Methods

### Patient recruitment

Approval from the SingHealth Centralised Institutional Review Board (CIRB Reference Number: 2017/2431) was obtained before patient recruitment commenced from 01 June 2017 to 01 December 2018. All aspects of the study, including patient recruitment and informed consent, were performed in accordance with the Declaration of Helsinki and the requirements of the SingHealth Centralised Institutional Review Board.

In this prospective cross-sectional study, healthy pregnant women in the first, second or third trimester who are patients at the KK Women’s and Children’s Hospital, and healthy non-pregnant women in KK Women’s and Children’s Hospital who are present for pre-conception or gynaecological consultations and satisfy the inclusion and exclusion criteria were identified by their doctors. The patients were approached and recruited in person on their routine visits or consultations at the KK Women’s and Children’s Hospital, and had their written informed consent taken by the investigators. A target of 200 healthy women between the ages of 21 and 45 years were recruited, comprising of 50 women who are not pregnant, and 50 pregnant women each in the first, second and third trimester with a single intrauterine pregnancy. Pregnant women who have been diagnosed with inevitable miscarriage, or women who have been known to have a history of recurrent miscarriages (3 or more consecutive pregnancy loss before 24 weeks of gestation), or women who have pre-existing luteal phase deficiency or other forms of diagnosed progesterone deficiency, or women who are planning to terminate pregnancy, or women with uncontrolled medical conditions, or women who have been treated with progesterone for any reasons in this current pregnancy, or women who have previous episodes of pregnancy related per vagina bleeding in this current pregnancy which may indicate threatened miscarriage were excluded.

Covariates for analysis, including maternal demographic, health, obstetric and lifestyle factors, were collected through an investigator-administered questionnaire. All participants were contacted and their case notes were checked to verify their successful delivery.

### Sample collection and quantification of progesterone and PIBF

10 ml of blood, taken via venipuncture, was collected from participants to measure serum progesterone and PIBF levels at presentation. Blood was collected into plain tubes and centrifuged for 10 min at 3000 g within 2 hours of collection. Serum samples were then stored at −80 degrees Celsius until analysis.

Serum progesterone levels were measured in the hospital’s clinical laboratory using the commercial Abbott ARCHITECT progesterone kit, which is a chemiluminescent microparticle immunoassay (CMIA), in accordance with the manufacturer’s protocol.

Serum PIBF concentrations were determined by an enzyme-linked immunosorbent assay (ELISA) using the Cusabio PIBF ELISA kit (CSB-E12872h, Cusabio Co. Ltd., China), which is a competitive enzyme immunoassay, in accordance with the manufacturer’s protocol. Serum samples from healthy non-pregnant women and healthy pregnant women in the first trimester were diluted 10 times from their original concentrations. Serum samples from healthy pregnant women in the second or third trimester were diluted 50 times from their original concentrations. All serum samples were diluted with phosphate-buffered saline (PBS). 50 μl of the diluted serum sample was added to the wells containing PIBF-specific antibodies and horseradish peroxidase (HRP)-conjugated PIBF, thereby initiating a competitive inhibition reaction by the PIBF-specific antibodies between PIBF in serum samples and HRP-conjugated PIBF. Subsequently, substrate solutions were added to the wells, causing the development of a coloured solution of which the optical density was negatively correlated with PIBF concentration. Absorbance was measured with a microplate reader (Synergy H1, Biotek, USA). Serum PIBF concentrations were quantified and validated using a standard curve of 90 kDa PIBF concentration against absorbance at 450 nm, which was produced using standards included in the manufacturer’s kit. Absorbance values were multiplied by the relevant dilution factor to obtain the original PIBF concentration.

### Statistical analysis

Baseline demographic data and biological markers of participants were summarised as mean ± SD for continuous variables and as percentages for categorical variables.

One-way analysis of variance (ANOVA) was used to compare groups of three or more continuous, normally distributed variables. Comparisons of categorical variables were accomplished using a chi-square test. Quantile regressions of progesterone (nmol/L) and PIBF (ng/ml) against gestational age (weeks) were used to determine the relationships of progesterone and PIBF with gestational age. Quantile regressions of progesterone (nmol/L) and PIBF (ng/ml) against BMI (kg/m^2^), segmented by trimesters, were used to investigate the relationships of progesterone and PIBF with BMI. Linear regression of ln(PIBF (ng/ml)) on ln(progesterone (nmol/L)) was performed to investigate the linear relationship between the two variables. SAS Studio 3.8 (Basic Edition) Software (SAS Institute Inc., Cary, NC, USA) was used for all statistical analyses. Statistical significance was set at p ≤ 0.05.

## Data Availability

All data generated or analysed during this study are included in this published article, unless otherwise stated.
